# Association between Three Waist Circumference-Related Obesity Metrics and Estimated Glomerular Filtration Rates

**DOI:** 10.3390/jcm11102876

**Published:** 2022-05-19

**Authors:** Dong Yoon Lee, Gyeong Im Yu, Yu-Mi Kim, Mi Kyung Kim, Min-Ho Shin, Mi-Young Lee

**Affiliations:** 1 Department of Preventive Medicine, Keimyung University School of Medicine, Daegu 42601, Korea; yardsticklee@gmail.com (D.Y.L.); rki0411@hanmail.net (G.I.Y.); 2 Department of Preventive Medicine, Hanyang University College of Medicine, Seoul 04763, Korea; kimyumi@hanyang.ac.kr (Y.-M.K.); kmkkim@hanyang.ac.kr (M.K.K.); 3 Department of Preventive Medicine, Chonnam National University Medical School, Gwangju 59626, Korea; mhshinx@hanmail.net

**Keywords:** body mass index, the residual method, chronic kidney disease, estimated glomerular filtration rate, obesity, waist circumference, waist-to-hip ratio, waist-to-height ratio, waist-to-height^0.5^ ratio

## Abstract

Studies that have assessed the associations between obesity and the estimated glomerular filtration rate (eGFR) have reported inconsistent results. This cross-sectional study was performed to investigate the associations between three waist circumference (WC)-related obesity metrics (waist-to-hip ratio (WHR), waist-to-height ratio (WHtR), and waist-to-height^0.5^ ratio (WHt.5R)) and eGFRs. This study included 2133 men and 3443 women who were older than 40 years with eGFRs ≥ 60 mL/min/1.73 m² from the Korean Multi-Rural Communities Cohort. We calculated the residual body mass index (BMI) to reduce multicollinearity among the obesity metrics and performed multiple linear regression. For both sexes, among the adjusted models, most of the general obesity metrics were significantly associated with eGFRs. Particularly for women, the WC-related and general obesity metrics had a stronger effect on eGFRs in the quartile models that included the BMI and the residual BMI, respectively. When WC-related obesity metrics had a stronger effect than the general obesity metric, for both sexes, WHtR showed a significant impact than WHt.5R and WHR on eGFRs. Reducing multicollinearity had an important role in assessing the obesity metrics’ association with eGFRs. Overall, applying the residual method in further studies might help with evaluating the obesity paradox on renal function.

## 1. Introduction

Obesity is a prevalent metabolic disease worldwide. In 2016, 650 million adults out of 1.9 billion had documented obesity cases [1]. Obesity is known as the leading cause of chronic kidney disease (CKD), and anthropometric indices, such as the body mass index (BMI), have been used to assess whether individuals are obese [2,3]. However, many studies have shown that the BMI may not be an efficient index for assessing WC-related obesity because it reflects the overall distribution of fat [4,5,6]. For instance, a study showed that 17% of men and 6% of women who were actually overweight in the general population would be missed when the BMI was solely used for screening [7].

The waist-to-hip ratio (WHR) and waist-to-height ratio (WHtR) are waist circumference (WC)-related indices. Several studies have shown that the risk of CKD is higher for those with WC-related obesity than for those with general obesity [8,9,10,11]. Although the WHR has been widely used as an index to measure WC-related obesity, it has the disadvantage of not reflecting the health status of Asians, who are small and/or thin when compared with Westerners; furthermore, the WHR does not include height in its equation [12,13]. Oh et al. (2013) reported that the WHtR was correlated with the eGFR; however, their study lacked representativeness because it targeted the residents of one small city and involved a small number of participants [9]. Moreover, previous studies that used the WHtR have focused on its association with cardiometabolic diseases. The waist-to-height^0.5^ ratio (WHt.5R) is a relatively newly established WC-related obesity metric [5,6,14,15,16]; however, its association with renal function has not yet been tested.

Performing comparisons between WC-related obesity metrics and the BMI may be important to evaluate which metrics show a significant impact on the eGFR when assessing renal function. However, the BMI does not perfectly represent general obesity and is not completely isolated from the proportion of WC-related obesity because of the BMI’s high correlation with the WHR, WHtR, and WHt.5R. According to previous studies, when the BMI and WC were assessed simultaneously in the same regression model, multicollinearity was indicated because they are strongly correlated [17]. In other words, the results could be biased by the multicollinearity between WC-related obesity metrics and the BMI, which might lead to inaccurate results. For instance, the residual WC was obtained by applying the residual method instead of by eliminating the highly correlated obesity metrics [18,19].

To our knowledge, this is the first study to assess the associations between eGFR and obesity metrics by applying the BMI or residual BMI to multiple linear regression. Using the Multi-Rural Communities Cohort Study (MRCohort), which represents three rural communities-based cohorts with a large number of participants, this study assessed whether the WC-related obesity metrics or general obesity metrics might have more significant associations with eGFRs. Moreover, we aimed to assess which WC-related obesity metrics show a significant impact on eGFRs.

## 2. Materials and Methods

### 2.1. Study Population

The community-based multicenter MRCohort was constructed as part of the Korean Genomic and Epidemiology Study. Given the data, baseline recruitment was performed from 2005 to 2010. A total of 9695 participants over the age of 40 who live in each rural community, such as Goryeong, Yangpyeong, and Namwon, responded to the on-site examinations. These rural areas were selected using a multi-stage cluster sampling method. Since our data were basically prospectively collected, the exclusion criteria were as follows: 2675 participants for one-time follow-up loss were excluded. Seven-hundred and eighty-one participants who had comorbidities such as myocardial infarction, cerebrovascular disease, or cancer at the baseline examination were excluded, as were participants who did not have records of laboratory data. Participants with missing records regarding lifestyle factors, such as tobacco use, alcohol consumption, regular exercise, and missing records of all other variables related to CKD were also excluded. Participants with a baseline eGFR of <60 mL/min/1.73 m² were excluded from the present study, and a total of 5576 participants were included in the study. The participants were analyzed by sex; there were 2133 men and 3443 women. One woman with a BMI ≥ 40 was considered an outlier and was excluded because bariatric surgery is recommended for morbid obesity (BMI ≥ 40) without Type 2 diabetes mellitus (DM), hypertension (HTN), or sleep apnea [20].

### 2.2. Data Collection

The examination procedures, including the anthropometric measurements, questionnaires, and clinical examinations were performed by trained personnel under the supervision of the Centers for Disease Control and Prevention National Research Institute of Health in Korea [21].

Data regarding demographics, lifestyle, disease, and medication histories were collected using a questionnaire. The identification number, age, sex, and educational status were collected as demographic information. The status of tobacco use, alcohol consumption, and regular exercise were collected as lifestyle information. All medical history data, including hypertension and diabetes mellitus were self-reported and recorded. Regarding anthropometric measurements, height was measured using a standard height scale and weight was measured after the scale was zero-balanced before each measurement. The BMI was calculated as weight (kg) divided by height (m)². WC and hip circumference (HC) were measured to the nearest 0.1 cm, and the range of WC was considered as the half between the lowest ribcage and the iliac crest during expiration; the range of HC was considered as the point of acetabular protrusion. Blood pressure was measured in the right arm at heart level after the participant had rested for 10 min. Two measurements were averaged over an interval of 5 min to obtain the systolic blood pressure and diastolic blood pressure from each subject. If the difference between the two measurements was more than 5 mmHg, the blood pressure was measured again. DM was defined as glucose level ≥ 126 with the use of antidiabetic medications or a diagnosis of DM before recruitment. HTN was defined as systolic blood pressure >140 mmHg and diastolic blood pressure >90 mmHg with the use of antihypertensive medications or a diagnosis of hypertension before recruitment. Laboratory tests were performed using blood samples collected after at least 8 h of overnight fasting. Triglyceride, low-density lipoprotein cholesterol (LDL-cholesterol), fasting glucose, serum uric acid, and creatinine levels were measured using an ADVIA 1650 automated analyzer (Siemens, New York, NY, USA).

### 2.3. Definition of Renal Functions

To assess the renal function, we used eGFRs obtained from the CKD-EPI equation, as this equation works well with an eGFR ≥ 60 mL/min/1.73 m² [22]. An eGFR of 60 to 89 mL/min/1.73 m² was defined as mild eGFR reduction; an eGFR ≥ 90 was defined as normal eGFR [23]. In the present study, eGFRs ≥ 60 mL/min/1.73 m² were used.

### 2.4. Definition of WC-Related Obesity Metrics

The WHR, WHtR, and WHt.5R, were defined as the WC-related obesity metrics in this study. The WHR was calculated as WC (cm)/HC (cm) and the WHtR was calculated as  WC (cm)/Height (cm). Finally, the WHt.5R was calculated as $\mathrm{WC}\;(m)/{\mathrm{Height}\;(m)}^{0.5}$ [4,5,6]. We used meters instead of centimeters in the WHt.5R equation to perform comparisons with the WHR and WHtR.

### 2.5. Statistical Analysis

Continuous variables are presented as mean ± standard deviation, and categorical variables are presented as frequencies and percentages. The Student’s *t*-test for continuous variables and the Chi-squared test for categorical variables were performed to compare differences between men and women. Stratification by sex was necessary because the body fat distributions within the two sexes morphologies differed. We analyzed the anthropometric characteristics of the study population by using STATA’s ‘xtile’ command, which categorizes participants into quartiles. Correlations were analyzed to ascertain multicollinearity among WHR, WHtR, WHt.5R, BMI, height, weight, WC, and HC.

Three WC-related obesity metrics were used to assess the renal function based on the eGFR. The WHR, WHtR, and WHt.5R (an independent variable in the WC-related obesity metrics) and their associations with eGFRs (dependent variable) were tested using multiple linear regression models with the BMI or residual BMI (an independent variable in the general obesity metrics). Before applying the residual method, the WC was omitted because of its strong correlation with the BMI, and WC-related ratios, including the WHR, WHtR, and WHt.5R, had to be tested using the same regression model with the BMI or residual BMI. The residual BMI, which was defined as the proportion of the BMI not related to the WC-related obesity metrics, was obtained by calculating the following equation: observed BMI−predicted BMI [24]. The residual BMI is not correlated with WC-related obesity metrics; in other words, the residual BMI represents the proportion of the BMI not explained by the WC-related obesity metrics, which includes height and/or WC in their equations (Figure S1). By using the residual BMI in the present study, the variance inflation factors (VIF) among independent variables were reduced, which led to stable standard errors and *p*-values.

Six different models were tested by comparing the BMI or residual BMI and WC-related obesity metrics. Model 1 (eGFR = BMI + WHR), Model 2 (eGFR = BMI + WHtR), and Model 3 (eGFR = BMI + WHt.5R) were tested without adjusting for confounders. Models 4, 5, and 6 used the same equations as Models 1, 2, and 3, and these were tested with their confounders being adjusted. With the residual BMI, Model 1 (eGFR = residual BMI + WHR),  Model 2 (residual BMI + WHtR), and Model 3 (eGFR = residual BMI + WHt.5R) were tested without their confounders being adjusted, and Models 4, 5, and 6 were tested with confounders adjusted. Moreover, the WC-related obesity metrics were divided into quartiles for comparisons with general obesity metrics. Six multiple models were tested with the BMI or residual BMI, and the equations were similar to those of the aforementioned models. Unstandardized coefficients and standard errors of the multiple linear regressions were used to assess which WC-related obesity metrics showed a significant impact on eGFR. Standardized beta coefficients were used to assess which obesity metrics had a stronger effect when compared with each other metrics. Multiple linear regression models were used to assess the contribution of obesity metrics to eGFRs by adjusting for covariates such as age (years), educational attainment (uneducated/elementary school/middle school/high school/college or more education), tobacco use (non-smoker/ex-smoker/current smoker), alcohol consumption (non-drinker/ex-drinker/current drinker), and regular exercise (no/yes); DM and HTN (categorical); triglycerides and low density lipoprotein-cholesterol (LDL-cholesterol) (continuous). Additionally, the effect modifications of sex and WC-related obesity metrics on eGFRs were performed to complement sex-stratification analyses.

All statistical analyses were conducted using STATA 16.1 (StataCorp LP, College Station, TX, USA). A two-tailed *p* < 0.05 was considered statistically significant.

## 3. Results

### 3.1. Characteristics of the Multi-Rural Communities Cohort

**Figure 1:**
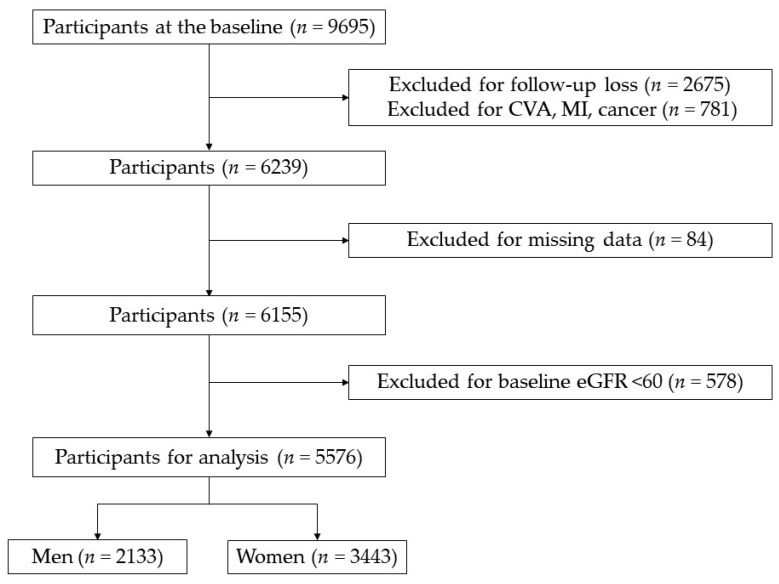
Flow diagram of the study population.

We analyzed 2133 men and 3443 women using the MRCohort (Figure 1). The general characteristics of the study population are presented according to sex in Table 1. Men and women had significant differences in all characteristics except for regular exercise and hypertension (*p* < 0.001). The mean age of the men was 61.41 ± 8.98, and that of the women was 59.65 ± 9.14 years. The BMI and WC were stratified, and the participants were evenly distributed in all BMI subgroups except the ≥30.0 subgroup. Of note, 43.6% of women had a WC of more than 85 cm and 23.4% of women had a WC more than 90 cm. The mean WHR values were 0.92 and 0.89 for men and women, respectively. The mean WHtR values were 0.52 and 0.55 for men and women, respectively. The mean WHt.5Rs were 0.67 and 0.68 for men and women, respectively. Men and women had similar rates of HTN (36.0% and 35.9%, respectively); however, the rate of DM was lower for women (9.0%) than for men (13.5%). The creatinine levels of women were lower than those of men. The results of the analysis of anthropometric characteristics are presented in Table S1. In Table S1, Quartile 4 (Q4) for height, weight, WC, WHR, and HC were found more frequently in men, whereas Q4 for BMI, WHtR, and WHt.5R were more frequent in women. Correlations between variables are presented in Table S2. 

### 3.2. Multiple Linear Regression of eGFR and Three Obesity Metrics Ratios Compared with the BMI or Residual BMI

We performed a multiple linear regression of the eGFR and three WC-related obesity metrics and general obesity metrics according to sex (Table 2 and Table 3). In Table 2, among the crude models, all the metrics in women were significantly associated with eGFRs (WC-related and general obesity: *p* < 0.05). For both sexes, the WHtR (men: B = −28.155; women: B = −30.737) showed a more significant impact than the WHR and WHt.5R on the eGFRs. Further, the WC-related obesity metrics had a stronger effect than the BMI (men: β = −0.144; women: β = −0.197). Consistently, among the adjusted models, the metrics in women were significantly associated with eGFRs (WC-related and general obesity: *p* < 0.05). For both sexes, the WHtR showed a more significant impact than the WHR and WHt.5R on eGFRs (men: B = 15.509; women: B = 16.528), Furthermore, the BMI (men: β = −0.202; women: β= −0.101) had a stronger effect than the WC-related obesity metrics. The VIFs of all models were less than four (men: 1.35–3.74; women: 1.17–2.87).

In Table 3, among the crude models, the metrics in women were significantly associated with the eGFR (WC-related and general obesity: *p* < 0.05), and WC-related obesity metrics were significantly associated with the eGFR for men (*p* < 0.05). For both sexes, the WHtR (men: B = −21.924; women: B = −13.819) showed a more significant impact than WHR and WHt.5R on eGFRs. For both sexes, particularly for men, the WC-related obesity metrics had a stronger effect than the residual BMI (men: β = −0.111; women: −0.090). Among adjusted models, the metrics, except for Model 4 in men and the general obesity metrics in women, were significantly associated with the eGFR (*p <* 0.05). The WHtR in men and women showed a more significant impact than other metrics (men: B = −16.040; women: B = 4.904) on eGFRs. For both sexes, most residual BMIs had a stronger effect than the WC-related obesity metrics (men: β = −0.137; women: β = −0.069). The VIFs of all models were assessed (men: 1.00–1.30; women: 1.00–1.22). 

The effect modifications of sex were additionally analyzed to complement the sex-stratification analyses (Tables S3 and S4). The effect modifications of sex and the WC-related obesity metrics were significantly associated with eGFR (*p* < 0.05). The VIFs of the interactions indicated multicollinearity, as well as the VIFs of the WC-related obesity metrics.

### 3.3. Multiple Linear Regression of eGFR and Quartiles of Three Obesity Metrics Ratios Compared with the BMI or Residual BMI

In Table 4, among the crude models, the results of WHtR and WHt.5R for men were significantly associated with the eGFR (*p* < 0.05), whereas most metrics for women (except Q2 in Models 1 and 3) were significantly associated with eGFR (*p* < 0.05). The WHtR for both sexes showed a more significant impact on eGFRs than WHR and WHt.5R (men: B = −1.512, −2.586, −4.311; women: B = −1.050, −2.671, −4.059). Thus, the eGFR is expected to decrease by −1.512, −2.586, and −4.311 mL/min/1.73 m² and −1.050, −2.671, and −4.059 mL/min/1.73 m² in men and women, respectively, with a unit increase in the WHtR. Most WC-related obesity metrics for both sexes had a stronger effect than the BMI (men: β = −0.070, −0.195, −0.198; women: β = −0.109, −0.186, −0.158). For both sexes, the dose–response association between each quartile of the WC-related obesity metrics is shown. Among the adjusted models, the results of the BMI for both sexes were significantly associated with the eGFR (*p* < 0.05). Q4 of WHR and WHt.5R (B = 0.783 and −0.463) for men showed a more significant impact on eGFRs than the quartiles of WHtR. Thus, eGFR is expected to increase by 0.783 mL/min/1.73 m² in Q4 of the WHR and decrease by −0.463 mL/min/1.73 m² in Q4 of the WHt.5R. The BMI for both sexes (except Q4 of the WC-related obesity metrics in women) had a stronger effect than the WC-related obesity metrics (men: β = −0.146, −0.146, −0.123; women: β = −0.036, −0.076, −0.065).

In Table 5, among the crude models, the results of the WC-related obesity metrics (except Q2 of WHR) for men were significantly associated with the eGFR (*p* < 0.05), whereas all metrics (except Q2 of the WC-related obesity metrics) for women were significantly associated with the eGFR (*p* < 0.05). In most models, Q4 of the WC-related obesity metrics showed a more significant impact on eGFR than any other quartiles (men: B = −2.009, −3.372, −3.315; women: B = −1.999, −2.083) (except for WHt.5R (−1.503) in women). Consequently, among most models, eGFR is expected to decrease by −2.009, −3.372, and −3.315 mL/min/1.73 m² and −1.999, −2.083, and −1.503 mL/min/1.73 m² in men and women, respectively. The WC-related obesity metrics for men had a stronger effect than the residual BMI (β = −0.091, −0.152, −0.150(Q4)), whereas the residual BMI of Models 2 and 3 for women had a stronger effect than the WC-related obesity metrics (β = 0.099, 0.089). Among the adjusted models, most results of both metrics (except for Q2 and Q4 of WHR, Q2 of WHtR) for men were significantly associated with the eGFR (*p* < 0.05), whereas the results of the residual BMI for women were significantly associated with the eGFR (*p* < 0.05). The WHt.5R (men: B = −1.224, −1.665, −2.903; women: B = 0.665, −0.403, 0.650) for both sexes showed a more significant impact on eGFRs than WHR and WHtR. Thus, the eGFR is expected to decrease by −1.224, −1.665, and −2.903 mL/min/1.73 m² in men, with a unit increase in the WHt.5R. However, the eGFR is expected to change by 0.665, −0.403, and 0.650 mL/min/1.73 m² in women, with a unit increase in the WHt.5R. And most models that included the residual BMI for both sexes (except for Model 6 in men and Model 4 in women) had a stronger effect than those that included the WC-related obesity metrics (men: β = −0.134, −0.122; women: β = −0.068, −0.053). The VIFs were kept at less than two. The dose–response relationship for men was observed in all models (except for Model 4), whereas non-linear relationships were observed for women (except for Model 5).

## 4. Discussion

This study assessed the WC-related obesity metrics and general obesity metrics that are associated with renal function, based on eGFRs. First, we compared both obesity metrics using WC-related obesity metrics and the BMI or residual BMI. Among the adjusted models, for both sexes, most of the general obesity metrics were significantly associated with the eGFR. Of note, it was shown that, particularly for women, the WC-related and general obesity metrics had a stronger effect on eGFRs in the quartile models that included the BMI and the residual BMI, respectively. Second, when the WC-related obesity metrics had a stronger effect than the general obesity metric, the WHtR of both sexes showed a more significant impact than WHR and WHt.5R on eGFRs. Third, reducing multicollinearity had an important role in assessing obesity metrics’ association with eGFRs.

Although our results could be varied depending on the model, the BMI and residual BMI were significantly associated with most of the models (except for crude models in men). BMI is known to be a gold-standard in diagnosing obesity. According to meta-analyses, an increased BMI was associated with a high risk of a low eGFR [25]. The incidence of CKD has been shown to increase as BMI trajectories increase [26]. Additionally, with the BMI being a protective factor, a multi-regional study demonstrated that lean-fat (low BMI/high WHtR) participants, mostly women, were vulnerable to worse outcomes such as mortality, although this study’s outcome was heart failure [27]. From these previous studies, diagnosing the obese using the BMI have been inconsistent. Since the BMI is calculated using weight (kg) divided by height (m) squared (weight represents general obesity and height represents WC-related obesity), Oh et al. (2013) reported that weight and height may not be associated with decreases in eGFRs. This phenomenon might be induced by relying on the BMI alone when assessing the associations with outcomes because the proportion of WC-related obesity metrics was not completely isolated from the BMI. Thus, using the BMI as the general obesity metric should be carefully considered; ultimately, studies using the residual BMI should be conducted in the future. Since the WC-related metrics of the crude models were partly significantly associated in women in the present study, several studies have reported that the associations between eGFRs and obesity metrics could vary based on the characteristics of the population. One study compared the WC with the BMI and indicated that the WC was a stronger risk factor for CKD, but only for young adults of 20–39 years of age [8]. Furthermore, studies involving a Korean cohort supported the hypothesis that all ages were important confounders that were involved in the associations among eGFRs and obesity metrics [8]. Moreover, a previous study reported a weak negative linear dose–response relationship between the WHtR and eGFR for non-menopausal women [28]. Consequently, when compared with the BMI or residual BMI, most of the WC-related obesity metrics were shown to be insignificant among the adjusted models in women. Thus, further investigations should be conducted particularly using a population of women.

During the present study, when the WC-related obesity metrics had a stronger effect than the general obesity metrics, the WHtR seemed to be the most relevant WC-related obesity metric. The WHtR might be predictive of low eGFRs; however, only the adjusted model was significantly associated with the CKD incidence for women [13]. Swainson et al. (2017) reported that the WHtR could be used as a predictor of the fat mass percentage and visceral adipose tissue mass, which have to be measured using total body dual-energy X-ray absorptiometry. Namely, the WHtR can be used as a stable metric in clinical practice, even without the use of instruments. Unlike the WHtR, the WHt.5R is a relatively new obesity metric that has weak effects on height and has a significant association with adiposity [6,29]. Regarding the association between eGFRs and the WHt.5R, WC, WHtR, and the WHt.5R were good predictors of the outcomes during metabolic studies of middle-aged and elderly Chinese populations [16]. The WHt.5R had the best predictive ability when identifying visceral adipose tissue, which could be observed with polycystic ovary syndrome [15]. Interestingly, two studies have insisted that the WHt.5R is the strongest predictor of cardiometabolic risk, and that the second strongest predictor is WHtR; other metrics included WC, BMI, and WHR [6,30]. Additionally, three other studies reported that the WHtR and WHt.5R were good predictors of outcomes [5,15,31]. In relation to these five studies, our results showed that the WHtR was the strongest WC-related obesity metric when compared with BMI or residual BMI for both sexes. In the present study, among the quartile models that included the residual BMI, the WHt.5R of the adjusted model in men had a strong effect, indicating a dose–response relationship. Unlike WHtR and WHt.5R, the WHR includes hip circumference in its equation. The WHR has been associated with renal function, particularly in the elderly population, due to BMI generally decreasing with age [32]. Additionally, the WHR is affected by sex and ethnicity, indicating an association with CKD in women [33,34,35]. A previous study demonstrated that the WHR of Black men was associated with eGFR decline, whereas the WHR of white men was not associated with eGFR decline. Additionally, White and Black women were associated with eGFR decline [12]. A meta-analysis pointed out that the HC might be an unstable index because it does not reflect changes in WC-related fat, especially in large populations [10]. Since the present study used the MRCohort, which represents three rural areas with a mean age of 60 in Korea, the impact of the WHR was shown to strongly affect the eGFR among the residual models, particularly in women.

The residual method was applied to each model (Table 3 and Table 5) [24]. Ngueta et al. (2013) and Zheng et al. (2021) used the residual method to obtain the residual WC from their regression models; in contrast to our study design, they investigated the associations among residual WCs and cardiometabolic factors. Thus, the results might vary between these two studies because of the characteristics of cohorts [18,19]. However, our study focused on the association with renal function based on eGFRs, and we obtained the residual BMI from each WHR, WHtR, and WHt.5R model. Testing these by using the same regression models enabled our study to assess realistic results by reducing multicollinearity. Collinearity can be determined using VIFs. Collinearity is considered when the VIF is greater than five; however, the VIF thresholds may differ according to the independent variables used in the study [17,36]. As the aforementioned studies have indicated, correlations among independent variables (>0.7) should be considered when the possibility of multicollinearity exists. In Table S2, correlations between the BMI and the WHtR or the WHt.5R for men were 0.7916 and 0.8202, respectively, and correlations between the BMI and the WHtR or the WHt.5R for women were 0.7342 and 0.7622, respectively. Although the VIFs of models that included the BMI in Table 2 and Table 4 remained less than four, our results in Table 3 and Table 5 were stabilized and became consistent; in other words, the cutoff values of the VIFs should be considered in accordance with the independent variables. Moreover, we additionally analyzed the effect modifications of sex and the WC-related obesity metrics on eGFRs. By designating men as the reference group, we could observe that each WC-related obesity metric that interacted with sex was significantly associated among adjusted models, ascertaining significant differences between results in both men and women. Of note, among the adjusted models that included the residual BMI, WHt.5R showed a significant impact on eGFR independently; however, the interaction between WHtR and women was shown to be strongest on eGFR. Since studies that use the residual BMI have not been published yet, our results of effect modifications cannot be compared. Zheng et al. (2021) once reported that Chinese adults were shown to be more susceptible to the effects of general obesity than any other ethnicities. Interestingly, in the present study, the VIFs of the WC-related obesity metrics were three to four, even when we tested with the residual BMI. It seems that using the residual BMI to reduce multicollinearity would not work in models of the effect modification.

The major strength of this study is that it is the first investigation to assess the associations between the eGFR and WC-related obesity metrics by applying residual methods to reduce multicollinearity, so that comparisons between WC-related obesity and general obesity on their association with eGFR could be performed. According to our results, WC-related obesity metrics could be simple and effective screening tools, even without the use of total body dual-energy X-ray absorptiometry [4,5,37]. The MRCohort is the largest population to date to be studied in order determine the associations between eGFRs and obesity metrics. Consequently, the MRCohort might represent the most general and anthropometric characteristics of individuals in rural areas in Korea. This study had a few limitations. With the MRCohort being a multi-rural cohort in a Korean population, our results cannot be generalized to other population or other ethnicities. Moreover, since our study population was originally designed to conduct the longitudinal study, further studies should be investigated to compare future results directly with this preliminary study. Several important risk factors that are critical in determining eGFR or the prevalence of CKD, such as the menopausal status, economic status, and cognitive status were unavailable in the given data. In particular, the menopausal status may be important for assessing associations with metabolic abnormalities in elderly women [38]. It was shown that the WHtR and BMI in postmenopausal populations are associated with changes in hemostatic factors and are particularly strongly associated with fibrinogen [39]. The eGFR was somewhat skewed according to the normality line; however, we did not transform the eGFRs into natural logarithms because the participants included 2133 and 3443 men and women, respectively, which may have conformed to the central theorem. Additionally, since this was a cross-sectional study, causality should not be inferred.

## 5. Conclusions

In conclusion, among the adjusted models for both sexes, most general obesity metrics were significantly associated with eGFR. When the WC-related obesity metrics had a stronger effect than the general obesity metric, the WHtR showed a more significant impact than WHR and WHt.5R on eGFRs. Reducing the multicollinearity had an important role in assessing the stable results of the association with eGFRs; therefore, we could ascertain that a VIF of less than four might indicate multicollinearity. The results of the present study may complement the results of previous studies by evaluating the obesity paradox on renal function based on eGFR. Furthermore, longitudinal studies that apply the residual method should be conducted.

## Figures and Tables

**Table 1:** General characteristics of the study populations by sex.

Characteristics	Men (*n* = 2133)	Women (*n* = 3443)	*p*-Value
Age (years)			
Mean ± SD	61.41 ± 8.98	59.65 ± 9.14	<0.001
Age groups, *n* (%)			<0.001
40–49	256 (12.0)	556 (16.2)	
50–59	588 (27.6)	1082 (31.4)	
60–69	869 (40.7)	1281 (37.2)	
70+	420 (19.7)	524 (15.2)	
Educational attainment, *n* (%)			<0.001
Uneducated	145 (6.8)	810 (23.5)	
Elementary school	910 (42.7)	1691 (49.1)	
Middle school	440 (20.6)	447 (13.0)	
High school	439 (20.6)	373 (10.8)	
College or higher	199 (9.3)	122 (3.6)	
Tobacco use, *n* (%)			<0.001
Non-smoker	663 (31.1)	3314 (96.3)	
Ex-smoker	796 (37.3)	46 (1.3)	
Current smoker	674 (31.6)	83 (2.4)	
Alcohol consumption, *n* (%)			<0.001
Non-drinker	482 (22.6)	2276 (66.1)	
Ex-drinker	216 (10.1)	93 (2.7)	
Current drinker	1435 (67.3)	1074 (31.2)	
Regular exercise, *n* (%)			0.741
No	1481 (69.4)	2405 (69.9)	
Yes	652 (30.6)	1038 (30.1)	
Obesity metrics			
BMI (kg/m²)	24.04 ± 2.95	24.57 ± 3.16	<0.001
−22.9	803 (37.7)	1115 (32.4)	<0.001
23.0–24.9	538 (25.2)	883 (25.7)	
25.0–29.9	751 (35.2)	1261 (36.6)	
30.0+	41 (1.9)	184 (5.3)	
Waist circumference (cm)	85.77 ± 8.28	83.46 ± 8.94	<0.001
Male: ≥90 Female: ≥85	673 (31.6)	1500 (43.6)	
Male: ≥95 Female: ≥90	290 (13.6)	807 (23.4)	
WHR	0.92 ± 0.06	0.89 ± 0.07	<0.001
WHtR	0.52 ± 0.05	0.55 ± 0.06	<0.001
WHt.5R	0.67 ± 0.06	0.68 ± 0.07	<0.001
eGFR (mL/min/1.73 m²)	77.98 ± 9.59	76.40 ± 9.45	<0.001
Hypertension	767 (36.0)	1235 (35.9)	0.946
Diabetes mellitus	288 (13.5)	311 (9.0)	<0.001
Total cholesterol (mg/dL)	191.89 ± 34.61	204.43 ± 35.86	<0.001
LDL-C (mg/dL)	116.02 ± 33.44	130.16 ± 32.66	<0.001
TG (mg/dL)	160.90 ± 110.43	144.03 ± 85.93	<0.001
HDL-C (mg/dL)	43.69 ± 11.10	45.47 ± 10.03	<0.001
HS-CRP (mg/L)	2.11 ± 5.09	1.54 ± 3.76	<0.001
Glucose (mg/dL)	104.27 ± 28.63	97.77 ± 18.76	<0.001
Creatinine (mg/dL)	1.04 ± 0.11	0.84 ± 0.08	<0.001
Albumin (g/dL)	4.46 ± 0.25	4.45 ± 0.23	0.058
Uric acid (mg/dL)	5.67 ± 1.41	4.27 ± 1.00	<0.001

BMI = the body mass index; WHR = waist-to-hip ratio; WHtR = waist-to-height ratio; WHt05R = waist-to-height^0.5^ ratio; eGFR = estimated glomerular filtration rate; LDL-C = low-density lipoprotein-cholesterol; TG = triglyceride; HDL-C = high density lipoprotein-cholesterol; HS-CRP = high-sensitivity C-reactive protein. Continuous variables are presented as means ± standard deviations, and categorical variables are presented as frequencies and percentages. *p*-value < 0.05 was considered significant.

**Table 2:** Multiple linear regression of estimated glomerular filtration rates and waist circumference-related obesity metrics, compared with the body mass index.

Model *	Men				Women			
	Coefficient (S.E.)	β	*p*-Value	VIF	Coefficient (S.E.)	β	*p*-Value	VIF
Model 1								
WHR	−8.167 (4.248)	−0.048	0.055	1.35	−14.609 (2.527)	−0.106	0.000	1.17
BMI	−0.157 (0.082)	−0.048	0.054	1.35	0.129 (0.055)	0.043	0.018	1.17
Model 2								
WHtR	−28.155 (6.967)	−0.142	0.000	2.68	−30.737 (3.885)	−0.197	0.000	2.17
BMI	0.130 (0.115)	0.040	0.259	2.68	0.440 (0.074)	0.147	0.000	2.17
Model 3								
WHt.5R	−22.238 (5.809)	−0.144	0.000	3.06	−23.143 (3.415)	−0.177	0.000	2.39
BMI	0.147 (0.123)	0.045	0.229	3.06	0.412 (0.078)	0.138	0.000	2.39
Model 4								
WHR	7.809 (4.051)	0.046	0.054	1.50	6.267 (2.439)	0.046	0.010	1.35
BMI	−0.519 (0.082)	−0.160	0.000	1.66	−0.118 (0.052)	−0.039	0.023	1.30
Model 5								
WHtR	15.509 (6.923)	0.078	0.025	3.21	16.528 (4.033)	0.106	0.000	2.87
BMI	−0.656 (0.120)	−0.202	0.000	3.54	−0.302 (0.075)	−0.101	0.000	2.69
Model 6								
WHt.5R	5.194 (5.604)	0.034	0.354	3.45	9.941 (3.378)	0.076	0.003	2.87
BMI	−0.534 (0.123)	−0.164	0.000	3.74	−0.242 (0.076)	−0.081	0.002	2.79

Coefficient (B), unstandardized coefficient; S.E., standard errors; β, standardized beta coefficient; VIF, variance inflation factors; WHR, waist-to-hip ratio; WHtR, waist-to-height ratio; WHt.5R, waist-to-height^0.5^ ratio; BMI, the body mass index. * Models 1, 2, 3 were crude models, and Models 4, 5, 6 were adjusted for confounders including age (continuous); educational attainment, tobacco use, alcohol consumption, regular exercise (categorical); diabetes mellitus and hypertension (categorical); triglycerides and low-density lipoprotein cholesterol (continuous). *p*-value < 0.05 was considered significant.

**Table 3:** Multiple linear regression of estimated glomerular filtration rates and waist circumference-related obesity metrics, compared with the residual body mass index.

Model *	Men				Women			
	Coefficient (S.E.)	β	*p*-Value	VIF	Coefficient (S.E.)	β	*p*-Value	VIF
Model 1								
WHR	−12.334 (3.658)	−0.073	0.001	1.00	−12.328 (2.334)	−0.090	0.000	1.00
Residual BMI	−0.157 (0.082)	−0.042	0.054	1.00	0.129 (0.055)	0.040	0.018	1.00
Model 2								
WHtR	−21.924 (4.257)	−0.111	0.000	1.00	−13.819 (2.638)	−0.089	0.000	1.00
Residual BMI	0.130 (0.115)	0.024	0.259	1.00	0.440 (0.074)	0.100	0.000	1.00
Model 3								
WHt.5R	−16.505 (3.324)	−0.107	0.000	1.00	−9.394 (2.211)	−0.072	0.000	1.00
Residual BMI	0.147 (0.123)	0.026	0.229	1.00	0.412 (0.078)	0.089	0.000	1.00
Model 4 *								
WHR	−5.933 (3.508)	−0.035	0.091	1.12	4.188 (2.257)	0.030	0.064	1.15
Residual BMI	−0.519 (0.082)	−0.137	0.000	1.23	−0.118 (0.052)	−0.036	0.023	1.11
Model 5 *								
WHtR	−16.040 (4.103)	−0.081	0.000	1.13	4.904 (2.600)	0.031	0.059	1.19
Residual BMI	−0.656 (0.120)	−0.123	0.000	1.32	−0.302 (0.075)	−0.069	0.000	1.24
Model 6 *								
WHt.5R	−15.554 (3.231)	−0.101	0.000	1.15	1.872 (2.130)	0.014	0.380	1.14
Residual BMI	−0.534 (0.123)	−0.094	0.000	1.23	−0.242 (0.076)	−0.052	0.002	1.17

Coefficient (B), unstandardized coefficient; S.E., standard errors; β, standardized beta coefficient; VIF, variance inflation factors; WHR, waist-to-hip ratio; WHtR, waist-to-height ratio; WHt.5R, waist-to-height^0.5^ ratio; residual BMI, the residual body mass index. * Models 1, 2, 3 were crude models, and Models 4, 5, 6 were adjusted for confounders including age (continuous); educational attainment, tobacco use, alcohol consumption, regular exercise (categorical); diabetes mellitus and hypertension (categorical); triglycerides and low-density lipoprotein cholesterol (continuous). *p*-value < 0.05 was considered significant.

**Table 4:** Quartile of three waist circumference-related obesity metrics and its association with estimated glomerular filtration rates, compared with the body mass index.

Model *	Men				Women			
	Coefficient (S.E.)	β	*p*-Value	VIF	Coefficient (S.E.)	β	*p*-Value	VIF
Model 1								
WHR								
Q2	−0.205 (0.600)	−0.009	0.732	1.57	−0.832 (0.462)	−0.038	0.071	1.55
Q3	−1.539 (0.628)	−0.069	0.014	1.73	−2.190 (0.475)	−0.100	0.000	1.65
Q4	−1.543 (0.665)	−0.070	0.021	1.94	−2.370 (0.487)	−0.109	0.000	1.73
BMI	−0.126 (0.081)	−0.039	0.119	1.32	0.124 (0.055)	0.041	0.024	1.17
Model 2								
WHtR								
Q2	−1.512 (0.626)	−0.068	0.016	1.73	−1.050 (0.475)	−0.048	0.027	1.65
Q3	−2.586 (0.706)	−0.117	0.000	2.20	−2.671 (0.509)	−0.122	0.000	1.90
Q4	−4.311 (0.853)	−0.195	0.000	3.21	−4.059 (0.607)	−0.186	0.000	2.70
BMI	0.158 (0.104)	0.049	0.129	2.23	0.334 (0.069)	0.112	0.000	1.84
Model 3								
WHt.5R								
Q2	−1.765 (0.637)	−0.080	0.006	1.79	−0.458 (0.476)	−0.021	0.336	1.66
Q3	−2.415 (0.724)	−0.109	0.001	2.31	−2.832 (0.518)	−0.130	0.000	1.97
Q4	−4.381 (0.907)	−0.198	0.000	3.62	−3.454 (0.629)	−0.158	0.000	2.90
BMI	0.173 (0.111)	0.053	0.119	2.51	0.317 (0.071)	0.106	0.000	1.99
Model 4								
WHR								
Q2	0.313 (0.547)	0.014	0.567	1.59	0.508 (0.421)	0.023	0.227	1.59
Q3	−0.213 (0.579)	−0.010	0.713	1.79	0.376 (0.443)	0.017	0.397	1.77
Q4	0.783 (0.630)	0.035	0.214	2.12	1.113 (0.464)	0.051	0.016	1.93
BMI	−0.475 (0.081)	−0.146	0.000	1.62	−0.108 (0.052)	−0.036	0.038	1.29
Model 5								
WHtR								
Q2	0.291 (0.583)	0.013	0.617	1.81	0.683 (0.438)	0.031	0.119	1.73
Q3	0.230 (0.666)	0.010	0.730	2.36	1.091 (0.488)	0.050	0.025	2.14
Q4	0.414 (0.831)	0.019	0.618	3.68	2.033 (0.606)	0.093	0.001	3.31
BMI	−0.477 (0.107)	−0.146	0.000	2.84	−0.226 (0.067)	−0.076	0.001	2.17
Model 6								
WHt.5R								
Q2	−0.257 (0.591)	−0.012	0.664	1.86	1.110 (0.435)	0.051	0.011	1.70
Q3	−0.056 (0.678)	−0.003	0.934	2.45	0.333 (0.486)	0.015	0.494	2.13
Q4	−0.463 (0.866)	−0.021	0.593	4.00	1.823 (0.609)	0.084	0.003	3.35
BMI	−0.401 (0.111)	−0.123	0.000	3.04	−0.195 (0.069)	−0.065	0.005	2.27

Coefficient (B), unstandardized coefficient; S.E., standard errors; β, standardized beta coefficient; VIF, variance inflation factors; WHR, waist-to-hip ratio; WHtR, waist-to-height ratio; WHt.5R, waist-to-height^0.5^ ratio; BMI, the body mass index. * Models 1, 2, 3 were crude models, and Models 4, 5, 6 were adjusted for confounders including age (continuous); educational attainment, tobacco use, alcohol consumption, regular exercise (categorical); diabetes mellitus and hypertension (categorical); triglycerides and low-density lipoprotein cholesterol (continuous). Quartile 1 (Q1) was designated as the reference group, and Q1 was not shown in the Table 4. *p*-value < 0.05 was considered significant.

**Table 5:** Quartile of three waist circumference-related obesity metrics and its association with estimated glomerular filtration rates, compared with the residual body mass index.

Model *		Men				Women		
	Coefficient (S.E.)	β	*p*-Value	VIF	Coefficient (S.E.)	β	*p*-Value	VIF
Model 1								
WHR								
Q2	−0.381 (0.585)	−0.017	0.515	1.50	−0.712 (0.454)	−0.033	0.117	1.51
Q3	−1.832 (0.586)	−0.083	0.002	1.50	−1.979 (0.455)	−0.091	0.000	1.51
Q4	−2.009 (0.585)	−0.091	0.001	1.50	−1.999 (0.453)	−0.092	0.000	1.50
Residual BMI	−0.142 (0.082)	−0.038	0.082	1.00	0.145 (0.055)	0.045	0.009	1.01
Model 2								
WHtR								
Q2	−1.150 (0.583)	−0.052	0.049	1.50	−0.334 (0.451)	−0.015	0.460	1.50
Q3	−1.980 (0.583)	−0.089	0.001	1.50	−1.436 (0.452)	−0.066	0.001	1.50
Q4	−3.372 (0.583)	−0.152	0.000	1.50	−2.083 (0.452)	−0.095	0.000	1.50
Residual BMI	0.138 (0.115)	0.026	0.228	1.00	0.435 (0.074)	0.099	0.000	1.00
Model 3								
WHt.5R								
Q2	−1.348 (0.583)	−0.061	0.021	1.50	0.280 (0.452)	0.013	0.536	1.50
Q3	−1.725 (0.583)	−0.078	0.003	1.50	−1.607 (0.452)	−0.074	0.000	1.50
Q4	−3.315 (0.583)	−0.150	0.000	1.50	−1.503 (0.452)	−0.069	0.001	1.50
Residual BMI	0.165 (0.123)	0.029	0.179	1.00	0.411 (0.078)	0.089	0.000	1.00
Model 4								
WHR								
Q2	−0.352 (0.535)	−0.016	0.511	1.53	0.401 (0.414)	0.018	0.333	1.55
Q3	−1.318 (0.541)	−0.060	0.015	1.57	0.191 (0.426)	0.009	0.654	1.63
Q4	−0.958 (0.556)	−0.043	0.085	1.65	0.796 (0.434)	0.037	0.066	1.69
Residual BMI	−0.508 (0.082)	−0.134	0.000	1.23	−0.117 (0.052)	−0.036	0.025	1.11
Model 5								
WHtR								
Q2	−0.813 (0.537)	−0.037	0.130	1.54	0.214 (0.413)	0.010	0.605	1.54
Q3	−1.605 (0.544)	−0.072	0.003	1.58	0.294 (0.423)	0.013	0.487	1.62
Q4	−2.389 (0.558)	−0.108	0.000	1.66	0.756 (0.440)	0.035	0.085	1.74
Residual BMI	−0.649 (0.119)	−0.122	0.000	1.32	−0.301 (0.075)	−0.068	0.000	1.24
Model 6								
WHt.5R								
Q2	−1.224 (0.539)	−0.055	0.023	1.55	0.665 (0.411)	0.030	0.106	1.52
Q3	−1.665 (0.547)	−0.075	0.002	1.60	−0.403 (0.419)	−0.018	0.336	1.58
Q4	−2.903 (0.561)	−0.131	0.000	1.68	0.650 (0.432)	0.030	0.132	1.68
Residual BMI	−0.514 (0.123)	−0.090	0.000	1.23	−0.246 (0.076)	−0.053	0.001	1.18

Coefficient (B), unstandardized coefficient; S.E., standard errors; β, standardized beta coefficient; VIF, variance inflation factors; WHR, waist-to-hip ratio; WHtR, waist-to-height ratio; WHt.5R, waist-to-height^0.5^ ratio; BMI, the body mass index. * Models 1, 2, 3 were crude models, and Models 4, 5, 6 were adjusted for confounders including age (continuous); educational attainment, tobacco use, alcohol consumption, regular exercise (categorical); diabetes mellitus and hypertension (categorical); triglycerides and low-density lipoprotein cholesterol (continuous). Quartile 1 (Q1) was designated as the reference group, and Q1 is not shown in the Table 4. *p*-value < 0.05 was considered significant.

## Data Availability

Data sharing is not applicable to this article.

## Supplementary Materials

The following supporting information can be downloaded at: https://www.mdpi.com/article/10.3390/jcm11102876/s1, Table S1: Anthropometric characteristics of the study populations by sex, Table S2: Correlation between estimated glomerular filtration rates and obesity metrics-related indices in both sexes, Table S3: The effect modification of sex on the link metrics and the body mass index, Table S4: The effect modification of sex on the link metrics and the residual body mass index, Figure S1: Scatterplots of the residual body mass index and three waist circumference-related obesity metrics in both sexes.
