# Supplementary material for: Association between Three Waist Circumference-Related Obesity Metrics and Estimated Glomerular Filtration Rates
Source: J Clin Med. 2022 May 19;11(10):2876. doi: 10.3390/jcm11102876 (PMC9147861; doi:10.3390/jcm11102876)
Supplement: Supplementary file 1 [file jcm-11-02876-s001.zip › jcm-1687136-supplementary.pdf]

List

Table S1. (Page 2)

Table S2. (Page 3)

Table S3. (Page 4)

Table S4. (Page 5)

Figure S1. (Pages 6–9)

Table S1. Anthropometric characteristics of the study populations by sex.

**Anthropometric Indices**	**Category**	**Men (*n* = 2133)**	**Women (*n* = 3443)**	***p*-Value**
Height (cm)		165.22±6.03	152.55±5.83	0.000
	Q1	26 (1.22%)	1379 (40.05%)	
	Q2	142 (6.66%)	1247 (36.22%)	
	Q3	676 (31.69%)	721 (20.94%)	
	Q4	1289 (60.43%)	96 (2.79%)	
Weight (kg)		65.79±9.94	57.28±8.66	0.000
	Q1	241 (11.30%)	1166 (33.87%)	
	Q2	346 (16.22%)	1047 (30.41%)	
	Q3	591 (27.71%)	792 (23.00%)	
	Q4	955 (44.77%)	438 (12.72%)	
BMI (kg/m²)		24.04±2.95	24.57±3.17	0.000
	Q1	591 (27.71%)	804 (23.35%)	
	Q2	524 (24.57%)	870 (25.27%)	
	Q3	551 (25.83%)	843 (24.48%)	
	Q4	467 (21.89%)	926 (26.90%)	
WC (cm)		85.77±8.28	83.47±8.94	0.000
	Q1	414 (19.41%)	987 (28.67%)	
	Q2	493 (23.11%)	897 (26.05%)	
	Q3	601 (28.18%)	809 (23.50%)	
	Q4	625 (29.30%)	750 (21.78%)	
WHR		0.92±0.06	0.89±0.07	0.000
	Q1	315 (14.77%)	1080 (31.37%)	
	Q2	536 (25.13%)	857 (24.89%)	
	Q3	625 (29.30%)	772 (22.42%)	
	Q4	657 (30.80%)	734 (21.32%)	
WHtR		0.52±0.05	0.55±0.06	0.000
	Q1	699 (32.77%)	697 (20.24%)	
	Q2	622 (29.16%)	771 (22.39%)	
	Q3	533 (24.99%)	861 (25.01%)	
	Q4	279 (13.08%)	1114 (32.36%)	
WHt.5R		6.67±0.62	6.76±0.72	0.000
	Q1	553 (25.93%)	842 (24.46%)	
	Q2	555 (26.02%)	839 (24.37%)	
	Q3	581 (27.24%)	813 (23.61%)	
	Q4	444 (20.81%)	949 (27.56%)	
HC (cm)		93.46±6.34	93.29±6.96	0.285
	Q1	523 (24.52%)	909 (26.40%)	
	Q2	528 (24.75%)	869 (25.24%)	
	Q3	538 (25.22%)	847 (24.60%)	
	Q4	544 (25.51%)	818 (23.76%)	

BMI, the body mass index; WC, waist circumference; WHR, waist-to-hip ratio; WHtR, waist-to-height ratio; WHt.5R, waist-to-height$^{0.5}$ ratio; HC, hip circumference. Categorical variables are presented as frequencies and percentages. Groups of quartiles were numbered. *P*-value < 0.05 was considered significant.

Table S2. Correlation between estimated glomerular filtration rates and obesity metrics-related indices in both sexes.

	eGFR	WHR	WHtR	WHt.5R	WC	BMI	Height	Weight	HC
(Men)									
eGFR	1.0000								
WHR	−0.0728*	1.0000							
WHtR	−0.1109*	0.7439*	1.0000						
WHt.5R	−0.1070*	0.7417*	0.9804*	1.0000					
WC	−0.0989*	0.7120*	0.9251*	0.9818*	1.0000				
BMI	−0.0729*	0.5085*	0.7916*	0.8202*	0.8174*	1.0000			
Height	0.0237	−0.0197	−0.1073*	0.0899*	0.2766*	0.1372*	1.0000		
Weight	−0.0486*	0.4015*	0.5883*	0.7073*	0.7958*	0.8764*	0.5932*	1.0000	
HC	−0.0711*	0.1222*	0.6466*	0.7288*	0.7811*	0.7035*	0.4104*	0.7685*	1.0000
(Women)									
eGFR	1.0000								
WHR	−0.0896*	1.0000							
WHtR	−0.0885*	0.7528*	1.0000						
WHt.5R	−0.0720*	0.7501*	0.9847*	1.0000					
WC	−0.0527*	0.7235*	0.9376*	0.9839*	1.0000				
BMI	0.0032	0.3815*	0.7342*	0.7622*	0.7666*	1.0000			
Height	0.1064*	−0.1483*	−0.2573*	- 0.0863*	0.0921*	0.0263	1.0000		
Weight	0.0571*	0.2520*	0.4952*	0.6053*	0.6992*	0.8635*	0.5222*	1.0000	
HC	0.167	0.0274	0.5845*	0.6537*	0.7039*	0.7147*	0.2844*	0.7503*	1.0000

eGFR, estimated glomerular filtration rates; WHR, waist-to-hip ratio; WHtR, waist-to-height ratio; WHt.5R, waist-to-height$^{0.5}$ ratio; WC, waist circumference; BMI, the body mass index; HC, hip circumference. Correlation > 0.7 between independent and dependent variables were assessed to have the multicollinearity. * p-value < 0.05

Table S3. The effect modification of sex on the link metrics and the body mass index

Model	Coefficient (S.E.)	β	*p*-Value	VIF
Model 1				
Intercept	89.298 (3.329)		0.000	
WHR	−13.338 (3.819)	−0.091	0.000	3.86
BMI	0.038 (0.046)	0.012	0.406	1.24
Women	−2.204 (3.963)	−0.112	0.578	230.50
WHR × Women	0.342 (4.338)	0.016	0.937	224.43
Model 2				
Intercept	89.685 (2.197)		0.000	
WHtR	−38.544 (5.175)	−0.234	0.000	5.62
BMI	0.346 (0.063)	0.112	0.000	2.34
Women	−6.919 (2.655)	−0.353	0.009	104.31
WHtR × Women	11.419 (5.017)	0.324	0.023	115.30
Model 3				
Intercept	89.627 (2.209)		0.000	
WHt.5R	−29.473 (4.175)	−0.213	0.000	5.15
BMI	0.333 (0.066)	0.108	0.000	2.60
Women	−7.537 (2.688)	−0.384	0.005	106.58
WHt.5R × Women	8.936 (3.990)	0.313	0.025	110.56
Model 4				
Intercept	121.047 (3.338)		0.000	
WHR	0.203 (3.523)	0.001	0.954	4.02
BMI	−0.242 (0.044)	−0.078	0.000	1.40
Women	−10.198 (3.614)	−0.520	0.005	234.45
WHR × Women	8.182 (3.958)	0.376	0.039	228.43
Model 5				
Intercept	124.564 (2.465)		0.000	
WHtR	2.315 (4.898)	0.014	0.636	6.13
BMI	−0.401 (0.063)	−0.130	0.000	2.94
Women	−12.674 (2.437)	−0.646	0.000	107.05
WHtR × Women	17.839 (4.584)	0.506	0.000	117.19
Model 6				
Intercept	125.875 (2.482)		0.000	
WHt.5R	−3.971 (3.893)	−0.029	0.308	5.46
BMI	−0.323 (0.065)	−0.105	0.000	3.07
Women	−14.079 (2.470)	−0.718	0.000	109.77
WHt.5R × Women	16.653 (3.655)	0.582	0.000	113.14

Coefficient (B), unstandardized coefficient; S.E., standard errors; β, standardized beta coefficient; VIF, variance inflation factors; WHR, waist-to-hip ratio; WHtR, waist-to-height ratio; WHt.5R, waist-to-height$^{0.5}$ ratio.

Model 1, 2, 3 were the crude models, and model 4, 5, 6 were the adjusted for confounders including age (continuous); educational attainment, tobacco use, alcohol consumption, regular exercise (categorical); diabetes mellitus (DM) and hypertension (categorical); triglycerides and low-density lipoprotein cholesterol (LDL-cholesterol) (continuous). When WC-related metrics x sex was calculated, men were designated as a reference. *P*-value < 0.05 was considered significant.

Table S4. The effect modification of sex on the link metrics and the residual body mass index.

Model	Coefficient (S.E.)	β	*p*-Value	VIF
Model 1				
Intercept	89.571 (3.346)		0.000	
WHR	−12.617 (3.639)	−0.086	0.001	3.50
Residual BMI	0.038 (0.046)	0.011	0.406	1.04
Women	−2.204 (3.963)	−0.112	0.578	230.50
WHR × Women	0.342 (4.338)	0.016	0.937	224.43
Model 2				
Intercept	90.714 (2.210)		0.000	
WHtR	−24.777 (4.243)	−0.150	0.000	3.78
Residual BMI	0.346 (0.063)	0.075	0.000	1.04
Women	−6.919 (2.655)	−0.353	0.009	104.31
WHtR × Women	11.419 (5.017)	0.324	0.023	115.30
Model 3				
Intercept	89.870 (2.213)		0.000	
WHt.5R	−17.756 (3.301)	−0.128	0.000	3.22
Residual BMI	0.333 (0.066)	0.067	0.000	1.01
Women	−7.537 (2.688)	−0.384	0.005	106.58
WHt.5R × Women	8.936 (3.990)	0.313	0.025	110.56
Model 4				
Intercept	119.307 (3.326)		0.000	
WHR	−4.395 (3.369)	−0.030	0.192	3.67
Residual BMI	−0.242 (0.044)	−0.072	0.000	1.18
Women	−10.198 (3.614)	−0.520	0.005	234.45
WHR × Women	8.182 (3.958)	0.376	0.039	228.43
Model 5				
Intercept	123.370 (2.448)		0.000	
WHtR	−13.653 (3.943)	−0.083	0.001	3.97
Residual BMI	−0.401 (0.063)	−0.087	0.000	1.30
Women	−12.674 (2.437)	−0.646	0.000	107.05
WHtR × Women	17.839 (4.584)	0.506	0.000	117.19
Model 6				
Intercept	125.639 (2.477)		0.000	
WHt.5R	−15.312 (3.077)	−0.110	0.000	3.41
Residual BMI	−0.323 (0.065)	−0.065	0.000	1.19
Women	−14.079 (2.470)	−0.718	0.000	109.77
WHt.5R × Women	16.653 (3.655)	0.582	0.000	113.14

Coefficient (B), unstandardized coefficient; S.E., standard errors; β, standardized beta coefficient; VIF, variance inflation factors; WHR, waist-to-hip ratio; WHtR, waist-to-height ratio; WHt.5R, waist-to-height$^{0.5}$ ratio.

Model 1, 2, 3 were the crude models, and model 4, 5, 6 were the adjusted for confounders including age (continuous), educational attainment, tobacco use, alcohol consumption, regular exercise (categorical) diabetes mellitus (DM) and hypertension (categorical); triglycerides and low-density lipoprotein cholesterol (LDL-cholesterol) (continuous). When

WC-related metrics x sex was calculated, men were designated as a reference. *P*-value < 0.05 was considered significant.

Figure S1. Comparison between the body mass index or the residual body mass index and three waist circumference-related obesity metrics presented in form of the scatterplots. Scatterplots of waist-to-hip ratio, waist-to-height ratio and waist-to-height0.5 ratio were presented in both men and women.

(a) WHR and BMI or residual BMI in both sexes

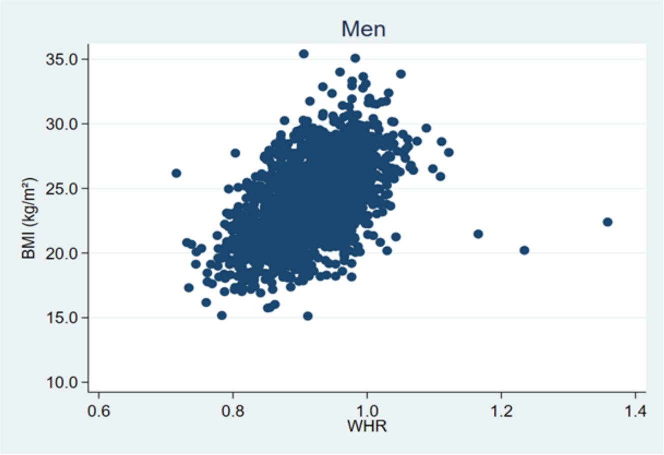

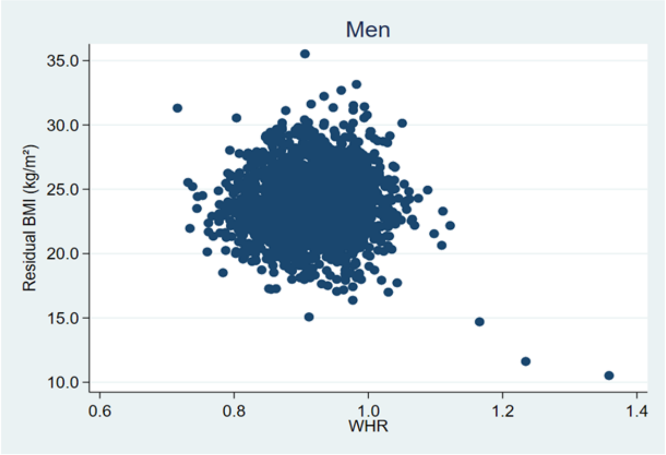

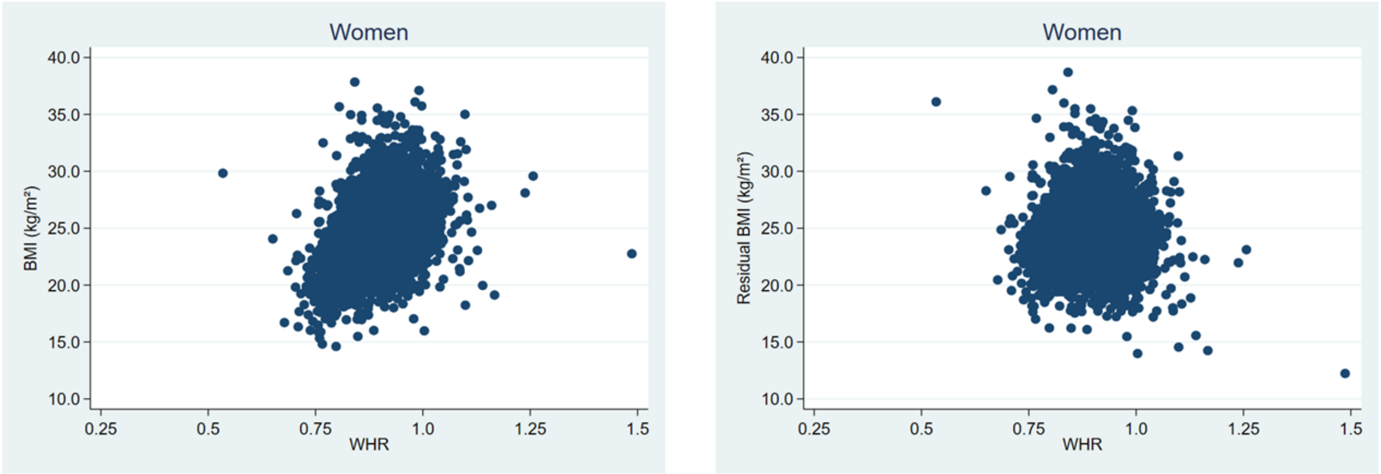

(b) WHtR and BMI or residual BMI in both sexes

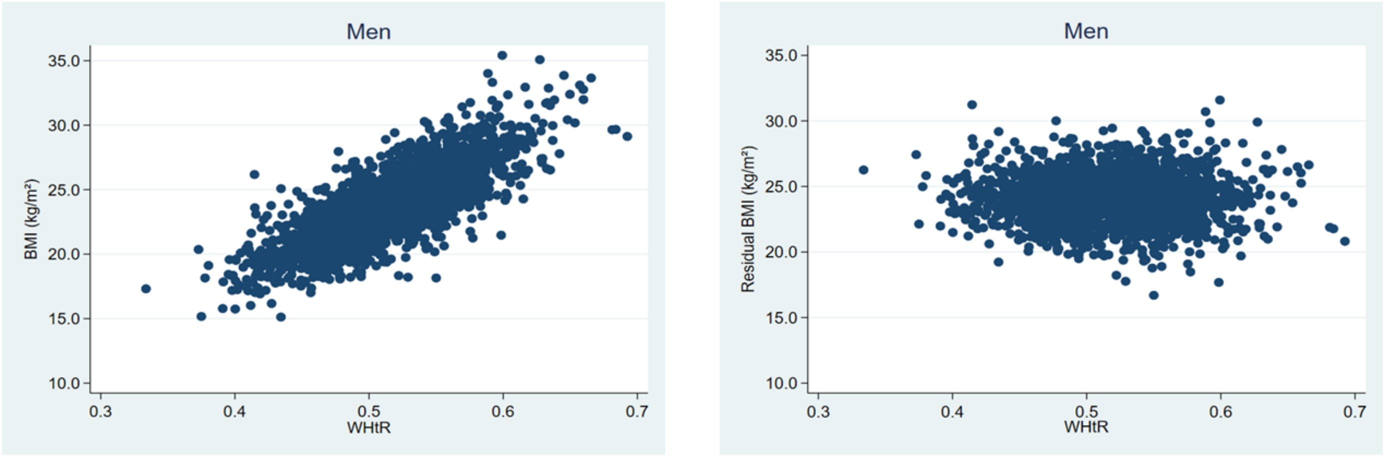

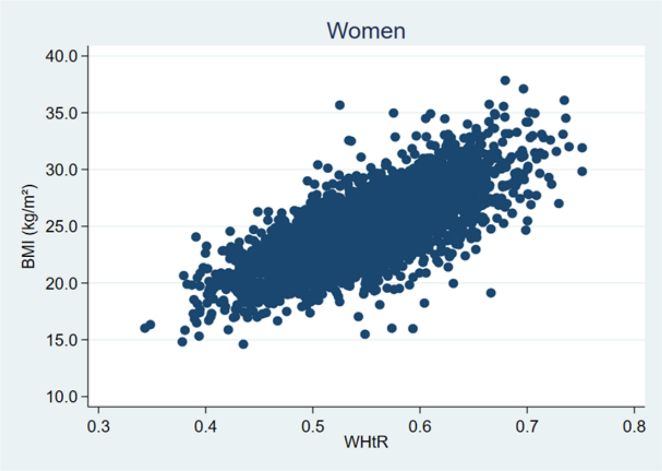

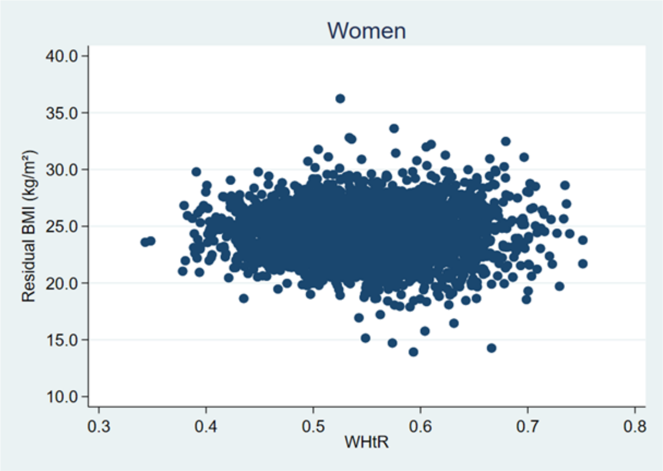

(c) WHt.5R and BMI or residual BMI in both sexes

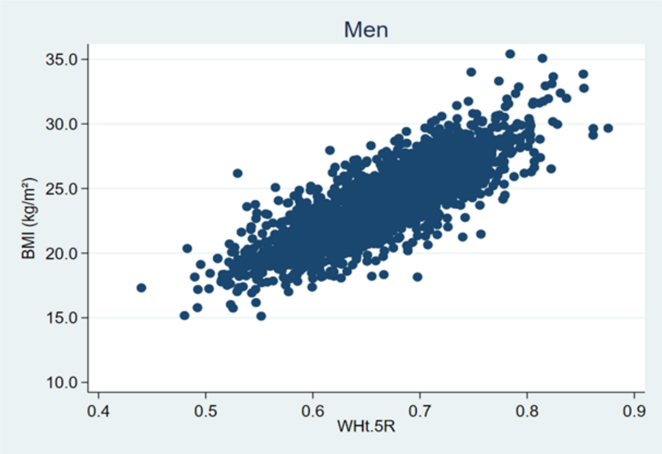

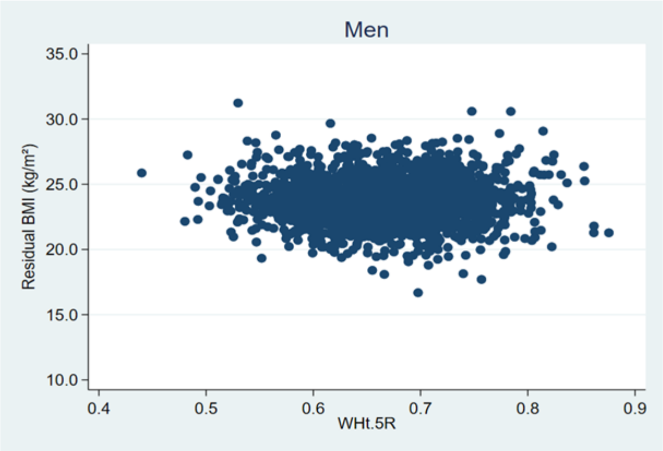

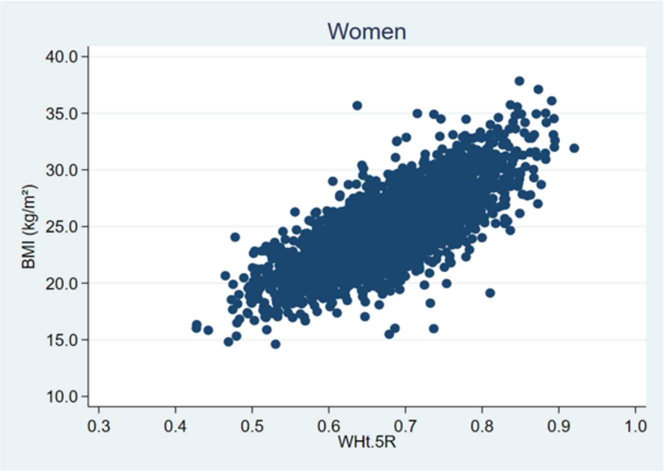
Women
BMI (kg/m²)
40.0
35.0
30.0
25.0
20.0
15.0
10.0
0.3
0.4
0.5
0.6
0.7
0.8
0.9
1.0
WHt.5R

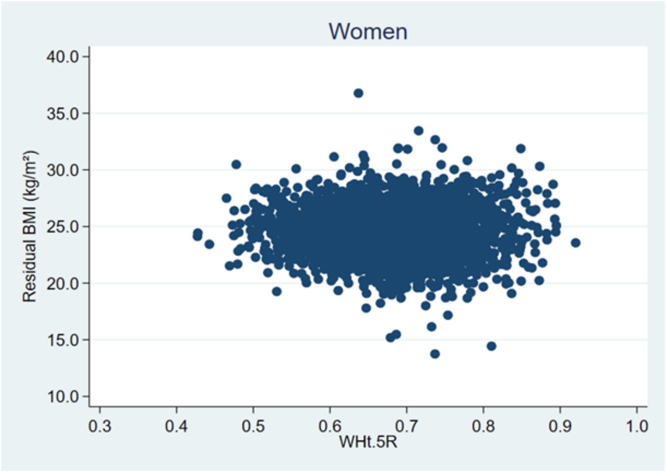
Women
Residual BMI (kg/m²)
40.0
35.0
30.0
25.0
20.0
15.0
10.0
0.3
0.4
0.5
0.6
0.7
0.8
0.9
1.0
WHt.5R
